# Soluble immune checkpoint-related proteins as predictors of tumor recurrence, survival, and T cell phenotypes in clear cell renal cell carcinoma patients

**DOI:** 10.1186/s40425-019-0810-y

**Published:** 2019-11-29

**Authors:** Qinchuan Wang, Jinhua Zhang, Huakang Tu, Dong Liang, David. W. Chang, Yuanqing Ye, Xifeng Wu

**Affiliations:** 1 Department of Surgical Oncology, Affiliated Sir Run Run Shaw Hospital and Department of Epidemiology and Health Statistics School of Public Health, Zhejiang University School of Medicine, Zhejiang, Hangzhou China; 2 Department of Epidemiology, The University of Texas MD Anderson Cancer Center, Houston, TX USA; 3 College of Life Science and Bioengineering, Beijing Jiaotong University, Beijing, China; 4 Department of Pharmaceutical Sciences, Texas Southern University, Houston, TX USA; 5 Department of Precision health and data science, School of Public Health and The Second Affiliated Hospital, Zhejiang University School of Medicine, 866 Yuhangtang Rd, Hangzhou, 310058 People’s Republic of China

**Keywords:** Soluble immune checkpoint-related proteins, Clear cell renal cell cancer, Predictor, Survival, Recurrence, Cytolytic activity

## Abstract

**Background:**

Immune checkpoint inhibitors have achieved unprecedented success in cancer immunotherapy. With the exception of a few candidate biomarkers, the prognostic role of soluble immune checkpoint-related proteins in clear cell renal cell cancer (ccRCC) patients is largely uninvestigated.

**Methods:**

We profiled the circulating levels of 14 immune checkpoint-related proteins panel (BTLA, GITR, HVEM, IDO, LAG-3, PD-1, PD-L1, PD-L2, Tim-3, CD28, CD80, CD137, CD27 and CTLA-4) and their associations with the risk of recurrence and death in 182 ccRCC patients using a multiplex Luminex assay. Gene expression in tumors from a subset of participating patients (*n* = 47) and another 533 primary ccRCC from TCGA were analyzed to elucidate potential mechanisms. Our primary endpoint is overall survival; secondary endpoint is recurrence-free survival. Multivariate Cox proportional hazard model, unconditional logistic regression model, and Kaplan-Meier analysis were applied in the study.

**Results:**

sTIM3 and sLAG3 were significantly associated with advanced (stage III) disease (*P* < 0.05). sPD-L2 was the strongest predictor of recurrence (HR 2.51, 95%CI 1.46–4.34, *P* = 9.33E-04), whereas high sBTLA and sTIM3 was associated with decreased survival (HR 6.02, 95%CI 2.0–18.1, *P* = 1.39E-03 and HR 3.12, 95%CI 1.44–6.75, *P* = 3.94E-03, respectively). Risk scores based on sTIM3 and sBTLA indicated that the soluble immune checkpoint-related proteins jointly predicted recurrence and death risks of ccRCC (*P* = 0.01 and 4.44E-04, respectively). Moreover, sLAG3 and sCD28 were found negatively correlated with cytolytic activity of T cells in tumors (rho = −0.31 and − 0.33, respectively).

**Conclusions:**

Our study provides evidence that soluble immune checkpoint-related proteins may associate with advanced disease, recurrence and survival in ccRCC patients, which highlights the prognostic values of soluble immune checkpoint-related proteins. Future independent validation in prospective studies is warranted.

## Introduction

Immunotherapy by immune checkpoint inhibition has achieved critical success in treating advanced clear cell renal cell carcinoma (ccRCC) during the past 5 years [1]; however, not all patients benefited from treatment. Although investigations of immune checkpoint genes and their products in RCC tumors have been conducted in the past, the prognostic role of soluble immune checkpoint markers has not been extensively explored, especially among localized cancer patients.

Previous studies have implicated high expression of inhibitory immune checkpoint genes in T cells strongly linked to T cell exhaustion and inefficient control of infections and tumors [2]. Giraldo et al. reported that in a group of 40 localized ccRCC cases the presence of CD8^+^PD-1^+^TIM3^+^LAG3^+^ tumor infiltrative lymphocytes (TILs) with CD4^+^ICOS^+^ T-reg cells identified patients with deleterious prognosis [3]. In a study involving 135 primary ccRCC cases and 51 metastatic ccRCC cases, PD-L2 expression in tumor cells and LAG3 expression in TILs were identified as poor prognostic factors in ccRCC patients [4]. In another cohort of RCC patients from Japan, high expression of immune checkpoint molecules in TILs correlated with poor overall and recurrence-free survival [5]. Thus, the expression of immune checkpoint genes in both immune and tumor cells may correlate with anti-tumor immunity in the tumor microenvironment (TME), pointing to the markers’ prognostic or therapeutic potential in ccRCC.

Only a few studies have examined the association between soluble immune checkpoint-related proteins and cancer outcomes. Circulating T cell regulatory proteins, some of which might modulate immune checkpoints, could be released from immune and tumor cells [6]. High level of circulating PD-L1 was associated with impaired immunity and poor outcomes in aggressive RCC, diffused large B-cell lymphoma and pancreatic cancer [7–9]. A spliced variant of PD-L1 was also reported to be secreted in the blood and to induce immune suppression in multiple cancers [10]. He Y et al. reported that low sLAG3 was associated with advanced stage in non-small-cell lung cancer (NSCLC) [11]. Taken together, these results suggest soluble immune checkpoint-related molecules may play a prognostic role in RCC and other cancers.

To identify soluble immune checkpoint-related proteins that can predict outcomes of ccRCC, patients, we implemented a three-stage strategy. First, we systematically assessed the level of soluble immune checkpoint-related proteins and their association with recurrence and survival in 182 ccRCC cases from an ongoing case-control study at The University of Texas MD Anderson Cancer Center (MDACC). Second, we evaluated the expression of immune genes in ccRCC tumors from the MDACC cohort, and we analyzed their association with cancer outcomes. Third, we analyzed immune gene expression in an external set of RCC tumor data from The Cancer Genome Atlas (TCGA). In short, this is an integrated, multi-stage investigation focus on peripheral immune checkpoint-related proteins and further supported by tumoral data from MDACC and TCGA cohorts.

## Materials and methods

### Study population and data collection

A schematic design of the study is shown in Additional file 1: Figure S1. ccRCC patients were drawn from an ongoing case-control study at MDACC (Houston, TX) initiated in 2002. The study has been approved by MDACC Institutional Review Board. Details of the study have been described previously [12]. In brief, all recruited cases were individuals with newly diagnosed (within 1 year of diagnosis), histologically confirmed ccRCC. All participants provided written informed consent before participating in the study. Patients’ clinical and follow-up data were abstracted from medical records. Epidemiologic data were collected by MDACC Staff interviewers through an in-person interview. Immediately after interview and consent, a 40 ml blood sample was collected in up to 5 Vacutainer tubes (Fisher Scientific, Waltham MA; consisting of 2 green top (sodium heparin), 1 red (no additive), 1 lavender (sodium EDTA), and 1 gold (gel clot activator)) from each participant and delivered to the laboratory. At the time of blood collection, all patients were previously untreated by surgery or chemotherapy. The plasma and peripheral blood monocytes (PBMC) were separated and stored in liquid nitrogen for further research. The tumor samples were snap frozen after they were taken from surgery and then stored in liquid nitrogen until processing. We selected only stages I-III and non-Hispanic white patients to minimize the effect of poor survival due to end-stage disease or metastasis and the confounding effect of population stratification, respectively. The study endpoints were overall survival and recurrence.

In addition, mRNA expression and clinical data (updated on 2016-01-28) for 533 primary ccRCC tumor samples with complete follow-up data were retrieved from TCGA using the Firebrowser portal (www.firebrowser.org).

### Detection of soluble immune checkpoint proteins in plasma

Plasma samples were assayed in duplicates using ProcartaPlex Human Immuno-Oncology Checkpoint Panel (Thermo Fisher, Waltham, MA) in 96-well plate format to quantify 14 human immune checkpoint markers. Assay was conducted according to protocols provided by the manufacturer using Luminex 200™ instrument and xPONENT® software (Luminex Corp, Austin, TX). In brief, 20 ul of plasma was used for each sample and mixed with ProcartaPlex Panel capture antibodies that are covalently bound to the surface of 6.5 μm microspheres dyed with precise proportions of red and infrared fluorophores to create unique spectral addresses that can be detected in the Luminex platform. Protein quantification is based on a fluorescently labeled secondary antibody whose signal intensity is proportional to the detected analyte concentration. A premixed antigen standard was serial diluted and applied as standard curve, and an inter-assay control was also used as positive control. Water or blank was used as negative control. After washing, fluorescent signals from all samples are detected in Luminex instrument, and data are analyzed using manufacturer provided software. All inter-assay and intra-assay coefficients of variation (CV) were below 15%. The Lower limits of quantification (LLOQ) of analyte was listed in Additonal file 1: Table S1.

### Tissue samples and MRNA extraction

All tissues were snap frozen in liquid nitrogen immediately after excision and stored at − 80 °C until use. Total RNA was extracted using Trizol Reagent (Thermo Fisher) according to the manufacturer’s instructions.

### Gene expression quantitation

Reverse transcription was performed using High-Capacity cDNA Reverse Transcription Kit according to manufacturer’s instructions (Thermo Fisher). Expression of genes *HAVCR2, CD28, CD27, CD80, CTLA4, BTLA, IDO1, PDCD1, CD274, PDCD1LG2, LAG3, TNFRSF9, TNFRSF14, TNFRSF4, PRF1, GZMA* and *GAPDH* were determined using TaqMan probes (Applied Biosystems, Waltham, MA) and Fluidigm 96.96 Dynamic Array (Fluidigm, San Francisco, CA), according to the manufacturer’s instructions. All probes were list in Additional file 1: Table S2.

### Statistical analysis

Recurrence free survival (RFS) was computed from the date of pathological diagnosis to the date of first documented local or distant recurrence or last follow-up death, whichever came first. Overall survival (OS) was defined as duration from diagnosis to death of any cause or last follow-up. Follow-up time is censored at the end of study or patient death, whichever comes first. The loss to follow-up patient was censored in this study. Levels of all soluble biomarkers and immune genes were dichotomized using a logistic regression spline model to generate better fit for non-linear data [13]. The cutoff point to determine high- and low-level groups was selected based on the smallest *P* value in the spline model. Comparison of host characteristics between subgroups was carried out using rank-sum test for continuous variables (age and BMI) and Pearson χ^2^ test for categorical variables (all other variables), For smoking history, never/former/current smoker was defined according to our previous study [14]. We estimated the association between each biomarker and risk of advanced ccRCC comparing early-stage (stage I and II) and late-stage (stage III) using the unconditional logistic regression model with adjustment for potential covariates including age, gender, smoking status, BMI, history of hypertension and diabetes. Risks of recurrence or death associated with each biomarker were analyzed using the multivariate Cox proportional hazard model with adjustment for the same covariates as listed above plus treatment, stage, grade and histology. A table listing the effects of covariates on the significance of association is shown in Additional file 1: Table S3. For the TCGA dataset with limited host information, only age, sex, stage and grade were adjusted for the analysis of death risk. To reduce the likelihood of false discovery, Bonferroni correction for multiple testing was also applied to *P* value of association. Differences in RFS and OS were assessed using the Kaplan-Meier survival analysis. Risk score was generated as a sum of the product of the dichotomized expression level of each significant marker by the beta coefficient in the Cox model. The risk score for survival was based on levels of sBTLA, sTIM3. All patients were dichotomized with the median value of the risk score into low- and high-risk groups. Cytolytic activity in tumors was calculated based on the geometric mean value of *GZMA* and *PRF1* expression [15]. Since *GZMB* is the most common granzyme in T cell activity, we also included alternative cytolytic activity calculation based on geometric mean of *GZMB* and *PRF1*. All statistical tests were two-sided with a significance cut-off at 0.05. All analyses were conducted using Stata 14.2 statistical software package (Stata Corp, College Station, TX).

## Results

### Patient characteristics

A total of 182 ccRCC cases were enrolled in this study including 90 early-stage (I and II) and 92 late-stage (stage III) patients. The demographic and clinical characteristics are listed in Table 1. There were no significant differences in host characteristics between early- and late-stage patients except for distribution of tumor grade and frequency of recurrence and death (*P* = 1.12E-07, 3.51E-16 and 0.04, respectively). Among all subjects, mean age was 59.0 years. Over two-thirds of the patients were males, and over half of them were smokers. A total of 80 patients (44.0%) were in obese status (BMI ≥ 30). A total of 91 (50%) patients had recurrent disease, while 33 (18.1%) patients had died. The median follow-up time (MFT) was 66.1 months (range: 1.1–134.1).

**Table 1:** Host characteristics

	ccRCC cases, *n* (%)	
Variables	Early stage^a^	Late stage^a^
Age, mean (SD)	58.46 (9.85)	59.57 (8.45)
BMI, mean (SD)	30.70 (6.39)	30.12 (7.07)
Sex		
Male	67 (74.44)	68 (73.91)
Female	23 (25.56)	24 (26.09)
Age		
< =60	44 (48.89)	43 (46.74)
> 60	46 (51.11)	49 (53.26)
Smoking status		
Never	40 (44.44)	43 (46.74)
Former	43 (47.78)	42 (45.65)
Current	7 (7.78)	7 (7.61)
BMI		
< 30	45 (50.00)	57 (61.96)
>= 30	45 (50.00)	35 (38.04)
Hypertension		
Yes	65 (72.22)	68 (73.91)
No	25 (27.78)	24 (26.09)
Diabetes		
Yes	19 (21.11)	17 (18.48)
No	71 (78.89)	75 (81.52)
Tumor grade^b^		
1	1	0 (0.00)
2	43 (47.78)	9 (9.78)
3	37 (41.11)	52 (56.52)
4	8 (8.89)	29 (31.52)
None	1 (1.11)	2 (2.17)
Recurrence		
Yes	17 (18.9)	74 (80.4)
No	73 (81.1)	18 (19.6)
Death		
Yes	11 (33.3)	22 (66.7)
No	79 (53.0)	70 (47.0)
Treatment		
Surgery-only	64 (70.33)	76 (83.52)
Surgery-plus-chemotherapy	27 (29.67)	15 (16.48)

^a^Early stage indicated stage I or II disease; late stage indicated stage III disease. The staging criteria derived from NCCN guidelines 2019 v.2.0

^b^Tumor grade based on Fuhrman criteria

### Soluble immune checkpoint-related proteins are associated with advanced disease

The Luminex multiplex assay was performed on all immune checkpoint-related proteins for early-stage and late-stage patients (Additional file 1: Table S4). Soluble CD137, HVEM, GITR, PD-1, and CD80 levels demonstrated minimal variations, thus these markers were not included in subsequent analyses.

We found that sLAG3 levels was increased in late-stage patients. Unconditional logistic regression analysis indicated that high level of sLAG3 (OR, 3.36, 95%CI 1.55–7.27, *P* = 0.002) were significantly associated with increased risk of advanced disease (Table 2).

**Table 2:** Soluble immune checkpoint proteins and association with clinical outcomes of ccRCC patients

Protein names	Advanced disease		Recurrence		Survival	
High vs low^a^	Adjusted OR (95%CI)^b^	*P* value	Adjusted HR (95%CI)^c^	*P* value	Adjusted HR (95%CI)^c^	*P* value
sTIM3	1 (reference)		1 (reference)		1 (reference)	
	2.61 (1.07–6.40)	0.04	1.65 (0.61–4.40)	0.32	**3.12 (1.44–6.75)**	**3.94E-03**^**#**^
sCD27	1 (reference)		1 (reference)		1 (reference)	
	0.62 (0.34–1.16)	0.14	1.44 (0.68–3.03)	0.34	2.80 (1.17–6.67)	0.02
sCD28	1 (reference)		1 (reference)		1 (reference)	
	0.45 (0.18–1.16)	0.10	2.30 (0.96–5.51)	0.06	2.71 (1.13–6.47)	0.02
sCTLA4	1 (reference)		1 (reference)		1 (reference)	
	1.75 (0.91–3.40)	0.10	2.13 (1.06–4.28)	0.03	1.12 (0.51–2.44)	0.78
sIDO	1 (reference)		1 (reference)		1 (reference)	
	1.25 (0.62–2.54)	0.54	0.48 (0.19–1.23)	0.13	0.49 (0.06–3.99)	0.51
sLAG3	1 (reference)		1 (reference)		1 (reference)	
	**3.36 (1.55–7.27)**	**2.13E-03**^**#**^	1.38 (0.32–6.00)	0.66	2.21 (0.95–5.15)	0.07
sBTLA	1 (reference)		1 (reference)		1 (reference)	
	0.66 (0.33–1.33)	0.25	0.61 (0.30–1.27)	0.19	**6.02 (2.00–18.1)**	**1.39E-03**^**#**^
sPDL1	1 (reference)		1 (reference)		1 (reference)	
	4.59 (0.91–23.3)	0.07	1.51 (0.89–2.56)	0.12	1.57 (0.70–3.49)	0.27
sPDL2	1 (reference)		1 (reference)		1 (reference)	
	0.53 (0.18–1.57)	0.25	**2.51 (1.46–4.34)**	**9.33E-04**^**#**^	2.39 (0.97–5.88)	0.06

Abbreviations: *OR* Odds ratio, *HR* Hazard ratio, *CI* Confidence interval. Significant values in bold font

^a^High- and low-level groups dichotomized by the logistic regression spline model [12]

^b^Adjusted by age, gender, smoking, BMI, diabetes, and hypertension

^c^Adjusted by age, gender, smoking, BMI, diabetes, hypertension, histology, grade, stage and treatment

# Significant after Bonferroni adjustment for multiple testing

### SOLUBLE IMMUNE CHECKPOINT-RELATED PROTEINS PREDICT ccRCC RECURRENCE AND OVERALL SURVIVAL

#### Recurrence

**Fig. 1:**
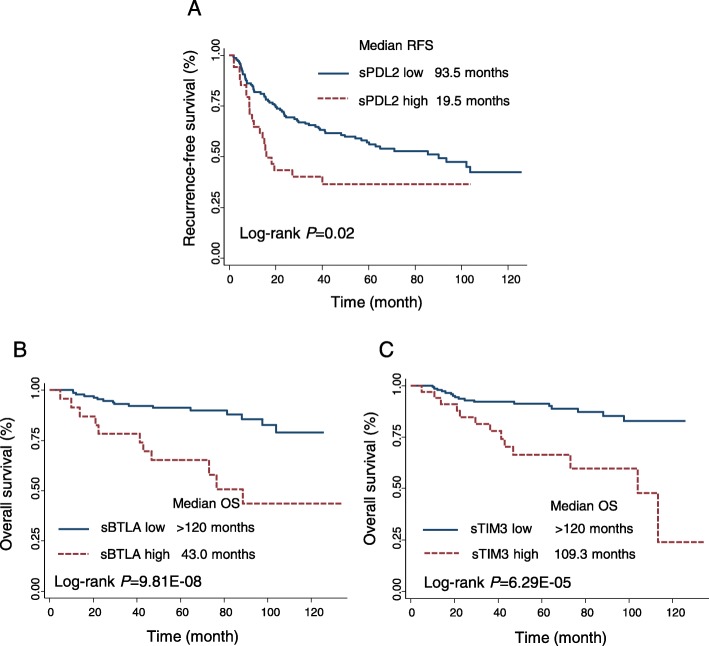
Kaplan Meier analysis of recurrence-free survival (RFS) and overall survival (OS) by levels of soluble immune checkpoint proteins in ccRCC patients. **a** Survival curve of RFS according to the level of sPDL2. **b-c** Survival curves of OS according to the levels of sBTLA, sTIM3, respectively. High- and low-level groups were dichotomized by the logistic regression spline model

Multivariate Cox proportional hazard analysis showed that patients with high level of sPD-L2 had significantly increased risk of recurrence (HR, 2.51, 95%CI 1.46–4.34, *P* = 9.33E-04), compare to low-level patients. Kaplan-Meier analysis indicated that high sPD-L2 levels were associated with decreased RFS (log-rank *P* = 0.02) (Fig. 1a, Table 2).

#### Overall survival (OS)

Multivariate Cox proportional hazard analysis demonstrated that sTIM3 and sBTLA were significantly associated with death risk in ccRCC patients. The most significant biomarker is sBTLA, patients with high sBTLA level had 6-fold increased death risk compare to patients with low sBTLA (95%CI 2.00–18.10, *P* = 1.4E-03). The OS was significantly reduced in high sBTLA patients (log-rank *P* = 9.81E-08) (Fig. 1b). sTIM3 was also significantly associated with death risk in ccRCC patients (HR = 3.12, 95%CI 1.44–6.75, *P* = 3.94E-03), the OS was significantly decreased in high sTIM3 patients (log-rank *P* = 6.29E-05) (Fig. 1c).

### ASSOCIATION OF sTIM3 WITH SURVIVAL IS DEPENDENT ON CLINICAL STAGE

Since sTIM3 was associated with both advanced (stage III) disease and survival, we investigated whether the association of sTIM3 with survival was dependent on clinical stage. In stratified analysis by early-stage (I and II) and late-stage (III) patients, we found sTIM3’s association with death risk was only significant in early-stage patients (HR = 36.1, 95%CI 3.73–350, *P* = 1.95E-03) but not in late-stage patients (HR = 1.62, 95%CI 0.60–4.42, *P* = 0.34) (Additional file 1: Table S5). Significant interaction was also found between sTIM3’s association with death risk and clinical stage (*P* = 0.007).

### PREDICTION OF ccRCC SURVIVAL BY RISK SCORE

**Fig. 2:**
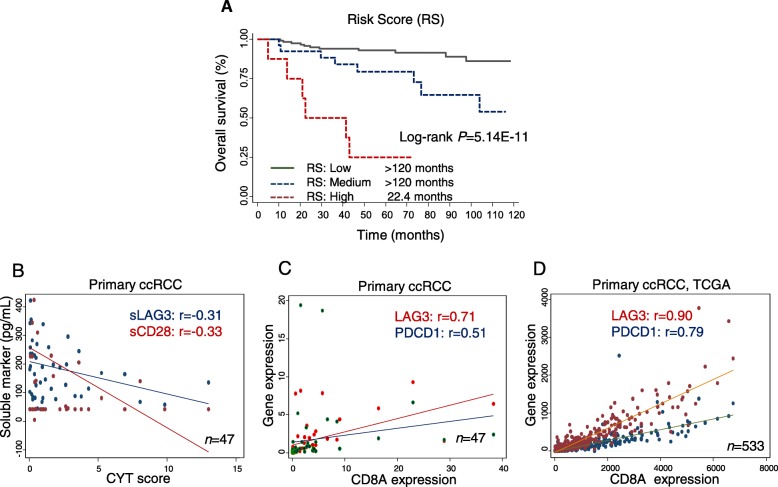
Risk score of soluble immune checkpoints as predictor of ccRCC survival and its correlation with T cell functions in primary tumors. **a** Risk score derived from 2 soluble immune checkpoint proteins (sBTLA, sTIM3) predicted overall survival (OS). Risk score green line represented low-risk group, and risk score blue dot line was medium-risk group, and red dot line was high-risk group. Risk groups were tertiled by the risk score. **b** Scatter plot of sLAG3 (blue) and sCD28 (red) levels (y-axis) against CYT score (x-axis). **c-d** Scatter plot of *LAG3* (red) and *PDCD1* (blue) expression (y-axis) against *CD8A* gene expression (x-axis) in ccRCC tumors from (C) MDACC cohort (*n* = 47) and (D) TCGA cohort (*n* = 533)

Based on levels of sTIM3 and sBTLA, a risk score for death risk was developed for all patients. Patients in the high-risk group and medium-risk group demonstrated increased risk of death (HR = 12.88, 95%CI 3.62–45.78, *P* = 7.88E-05; HR = 3.29, 95%CI 1.14–9.52, *P* = 0.028, respectively) compare to low-risk group patients. Kaplan-Meier survival analysis indicated that the OS was reduced in high-risk patients (log-rank *P* = 5.14E-11) (Fig. 2a, Additional file 1: Table S6).

### sLAG3 LEVEL CORRELATES WITH REDUCED INTRA-TUMOR CYTOLYTIC SCORE

To further validate our findings, we assessed the expression of immune genes in ccRCC tumors from TCGA database consisting of 533 primary ccRCC patients. We found that the expressions of all genes were higher in tumor tissues than corresponding normal tissues (*P* < 0.05) except for *CD274* (*PD-L1*) (Additional file 1: Figure S2).

Notably, we found that sLAG3 and sCD28 negatively correlated with T cell cytolytic score (rho = − 0.31 and − 0.33, *P* = 0.05 and 0.04, respectively) in our primary ccRCC tumors (Fig. 2b, Additional file 1: Table S6), whereas sPDL1 positively correlated with cytolytic score based on *GZMB* and *PRF1* expression (Additional file 1: Table S7). sLAG3 also negatively correlated with CD8A expression in tumors, while sPDL1 positively correlated with interferon gamma (*IFNG*) expression. We also demonstrated that the expression of *LAG3* and *PDCD1* in ccRCC tumors significantly correlated with *CD8A* expression in both MDACC cohort and TCGA cohort (Fig. 2c-d). The association between immune gene expression of ccRCC tumors and cancer outcomes were also analyzed in the MDACC cohort (*n* = 47) and TCGA cohort (*n* = 382); however, no associations were confirmed (Additional file 1: Table S8).

## Discussion

In this study, we identified a panel of soluble immune checkpoint-related proteins being associated with clinical outcomes of ccRCC patients. We demonstrated that circulating levels of sLAG3 are associated with risk of advanced disease; sPD-L2 level associated with risk of recurrence; sTIM3 and sBTLA levels correlated with risk of death in ccRCC patients. We generated a risk score combining the two biomarkers associated with survival indicating the soluble immune checkpoint-related proteins jointly predict the death risk of ccRCC. Furthermore, sLAG3 and sCD28 levels negatively correlated with the number and cytolytic activity of T cells in ccRCC tumors. These results highlighted the prognostic value of these soluble immune checkpoint-related proteins and unveiled potential biological mechanisms in ccRCC development.

We identified sPD-L2 as the most significant biomarker associated with ccRCC recurrence in this study. Previous work has described that sPD1 enhanced anti-tumor immunity by blocking PD-L1 in tumor cells [16], whereas sPD-L1 predicted poor prognosis in aggressive diffuse large B-Cell lymphoma [8]. In our study, sPD-L2 but not sPD-L1 was predictive of recurrence risk in ccRCC, and sPD-L2 level seems to be higher than that of sPD-L1, which is consistent with the protein expression of these two markers in TILs from another study [4]. This finding is partially supported by one previous study suggesting that high PD-L2 expression in tumor is associated with reduced cancer free survival in RCC patients [17]. The source of sPD-L2 may be derived from tumor exosomes [18] or alternatively activated macrophages [19] to inhibit T cell-mediated anti-tumor response. Therefore, high sPD-L2 could be a predictive biomarker of recurrence risk in ccRCC patients, although the finding warrants further confirmation in independent populations and exploration of the underlying biological mechanisms.

High density of LAG3+ T cells is a signature of T cell exhaustion in tumors [20]. Our results indicated that high level of sLAG3 is associated with advanced tumor stage in ccRCC patients. This is consistent with Camisaschi’s study that LAG3 were highly expressed in Treg cells in peripheral blood, tumor-involved lymph nodes and within tumor tissues isolated from patents with advanced (stage III and IV) melanoma and colorectal cancer [21]. Also, sLAG3 was also found marginally associated with poor survival (*P* = 0.07), which was supported by another study that sLAG3 was associated with poor survival in chronic lymphocytic leukemia, and sLAG3 could promote leukemic cell activation and anti-apoptotic effects [22]. However, another study of breast cancer showed that sLAG3 could serve as “Th1” (type I T helper cell) marker and that high level of sLAG3 predicted better OS [23]. This discrepancy may be due to the differential regulatory role of sLAG3 in mediating interaction between LAG3 and MHC-II, or the distinct immune landscapes of different cancer sites [20, 24]. Furthermore, we found that levels of sLAG3 negatively correlated with *CD8A* (T cell marker) expression and T cell cytolytic activity in tumors. Therefore, we propose that high level of sLAG3 may be indicative of T cell suppression in the TME, which in turn lead to advanced development of ccRCC.

TIM3 (*HAVCR2*) is an inhibitory receptor expressed on T cell and tumor cell surfaces which regulates Th1 and cytotoxic T cell responses [25, 26]. High TIM3 and PD1 expressions on T cells and in tumors are the signature of “deeply” exhausted status, which are frequently observed in ccRCC [27, 28]. The soluble TIM3 is shown to be generated by A Disintegrin and Metalloprotease (ADAM)-mediated ectodomain shedding from both T cells and tumor cells [29]. Our results revealed that sTIM3 is associated with advanced disease and increased death risk of ccRCC. This finding further confirmed previous studies that TIM3 + PD1+ TILs exhibited exhausted phenotype in the TME, thereby resulting in poor prognosis of cancer patients [3, 30]. The mechanisms of how sTIM3 interacts with its ligands, other inhibitory checkpoint proteins and TCR signaling pathway in T cells remain unclear necessitating further independent research. Interestingly, we found the association of sTIM3 with death risk was significant only in early-stage (I and II) patients suggesting the confounding effect of staging on survival, although our overall Cox proportional hazard analyses have been adjusted for clinical stage. The utility of sTIM3 as an early prognostic biomarker requires further confirmation in independent prospective studies.

BTLA is another inhibitory checkpoint protein that interacts with HVEM and LIGHT, a group of costimulatory molecules, resulting in suppression of T cell immunity [31]. In this study, sBTLA level (>2269 pg/mL) was identified as a predictor of poor OS in ccRCC patients indicating that sBTLA may play a similar role as membranous BTLA in suppressing T cell response. This is in line with Benjamin et al*’* study reported that sBTLA (>1910 pg/mL) could predict poor survival in pancreatic cancer patients [9]. BTLA expression in gastric cancer and lymphoma is also reported associate with poor prognosis [32, 33]. However, the immune function of sBTLA may varies by cancer sites, which warrants further research.

CTLA4 and CD28 play opposite roles during T cell activation [34]. However, in our study sCTLA4 and sCD28 were both associated with poor outcome of ccRCC patients, though not significant after multiple testing adjustment. sCTLA4 has been shown as an extrinsic suppressive factor of T cell activation, which could be prominently secreted by T-reg cells [35]. Our results revealed that sCTLA4 is associated with increased risk of recurrence. Meanwhile, sCD28 is also associated with death risk in our ccRCC cohort. We identified negative correlation between sCD28 and cytolytic activity in ccRCC tumors. This result is in line with one previous study showing sCD28 with inhibitory role in T cell proliferation in autoimmune diseases [36]. All of the above findings suggest the potential interactions between sCD28/sCTLA4 and anti-tumor immunity mediating their association with ccRCC outcomes.

There are several strengths to our study including the prospective high-quality cohort with relatively long follow-up time, the multiplex profiling of soluble immune checkpoint-related proteins and immune gene expressions in tumors, and the correlation analysis of soluble immune checkpoint-related proteins and T cell functions to provide biological validity. Despite these strengths, we also acknowledge some limitations. First, we have limited sample size with relatively small number of outcome events, which might constrain the power of our study. Limited tumor tissues available in our cohort and partial clinical outcome information in TCGA dataset might lead to some uncertainties in our results. Additional validation within larger independent cohort is necessary. After consideration of multiple testing, some of the associations we identified may not be significant. Nevertheless, the associations of sLAG3 with advanced (stage III) disease; sPD-L2 with recurrence; sTIM3 and sBTLA with survival remain significant after the stringent Bonferroni adjustment suggesting less likelihood for false discovery. Second, we did not conduct mechanistic studies to determine the functional impact of soluble immune checkpoint-related proteins. Instead, we evaluated the associations between soluble immune checkpoint-related proteins and T cell functional gene expressions to decipher potential mechanisms. Third, evaluation of immune checkpoint expression in peripheral blood leukocytes may be informative to examine any correlation between soluble protein levels and peripheral immune gene expression. Nevertheless, our inquiry is supported by one previous study correlating tumoral and peripheral blood T cell phenotypes with aggressiveness of ccRCC [3].

## Conclusion

In this study, we identified a panel of circulating immune checkpoint-related proteins that are associated with clinical outcomes and T cell phenotypes in ccRCC patients. Individually and jointly, the soluble immune checkpoint-related proteins may assist risk stratification of ccRCC patients to identify those at high risk of recurrence or poor survival for more intensive surveillance and/or treatment. Future study may apply these markers to test their predictive value for treatment outcome in immunotherapy-treated patients.

## Additional file

**Additional file1: Table S1.** Lower limits of quantification (LLOQ) of analyte. **Table S2.** Real-time PCR probes for T cell immune checkpoint gene expression used in this study. **Table S3.** Covariates of unconditional logistic regression model, multivariate Cox proportional hazard model for recurrence and survival. **Table S4.** Circulating levels of soluble immune checkpoint proteins in ccRCC patients. **Table S5.** Association with sTIM3 with death risk of ccRCC patients stratified by clinical stage. **Table S6.** Risk scores of soluble immune checkpoint biomarkers for ccRCC survival. **Table S7.** The correlation between circulating checkpoint level and T cell functions. **Table S8.** Tumor expression of immune checkpoint genes and association with ccRCC overall survival in the MDACC and TCGA cohorts. **Figure S1.** Schematic design of the study. **Figure S2.** Tumor and normal tissue expression of immune checkpoint genes in ccRCC patients derived from TCGA database. Maroon boxplot indicates tumor tissues (*N* = 533), and green boxplot indicates normal tissues (*N* = 72). Wilcoxon rank sum test was used in the comparison analysis. *indicates *P* < 0.05.

## Data Availability

The datasets used and/or analyzed during the current study are available from the corresponding author on reasonable request.

## Abbreviations

TIL: Tumor infiltrative lymphocyte
TME: Tumor micro-environment
