# Supplementary material for: Soluble immune checkpoint-related proteins as predictors of tumor recurrence, survival, and T cell phenotypes in clear cell renal cell carcinoma patients
Source: J Immunother Cancer. 2019 Nov 29;7:334. doi: 10.1186/s40425-019-0810-y (PMC6884764; doi:10.1186/s40425-019-0810-y)
Supplement: Supplementary file 1 — Additional file1: Table S1. Lower limits of quantification (LLOQ) of analyte. Table S2. Real-time PCR probes for T cell immune checkpoint gene expression used in this study. Table S3. Covariates of unconditional logistic regression model, multivariate Cox proportional hazard model for recurrence and survival. Table S4. Circulating levels of soluble immune checkpoint proteins in ccRCC patients. Table S5. Association with sTIM3 with death risk of ccRCC patients stratified by clinical stage. Table S6. Risk scores of soluble immune checkpoint biomarkers for ccRCC survival. Table S7. The correlation between circulating checkpoint level and T cell functions. Table S8. Tumor expression of immune checkpoint genes and association with ccRCC overall survival in the MDACC and TCGA cohorts. Figure S1. Schematic design of the study. Figure S2. Tumor and normal tissue expression of immune checkpoint genes in ccRCC patients derived from TCGA database. Maroon boxplot indicates tumor tissues (N = 533), and green boxplot indicates normal tissues (N = 72). Wilcoxon rank sum test was used in the comparison analysis. *indicates P < 0.05. [file 40425_2019_810_MOESM1_ESM.docx]

**Supplemental Materials**

**Supplemental Tables**

| **Supplemental Table S1. Lower limits of quantification (LLOQ) of analyte (data from ThermoFisher†).** | |
| --- | --- |
| **Analyte** | **LLOQ*** |
| BTLA | 144.65 |
| CD27 | 5.76 |
| CD28 | 42.02 |
| CD80 | 43.58 |
| CD137/4-1BB | 14.21 |
| CD152/CTLA4 | 9.52 |
| GITR | 27.2 |
| HVEM | 18.55 |
| IDO | 4.37 |
| LAG-3 | 11.21 |
| PD-1 | 7.13 |
| PD-L1 | 3.64 |
| PD-L2 | 48.71 |
| TIM-3 | 63.18 |
| * in pg/mL;  † ProcartaPlex Human Immuno-Oncology Checkpoint Panel, <https://www.thermofisher.com/order/catalog/product/EPX14A-15803-901> | |

| **Supplement table S2. Real-time PCR probes for T cell immune checkpoint gene expression used in this study** | | | |
| --- | --- | --- | --- |
| **Gene name** | **Official full name** | **Probe number** | **Catalog number** |
| *BTLA* | B and T lymphocyte associated (BTLA) | Hs00699198_m1 | 4331182 |
| *CD27* | CD27 molecule (CD27) | Hs00609654_g1 | 4351372 |
| *CD28* | CD28 molecule (CD28) | Hs00155512_m1 | 4331182 |
| *CD3E* | CD3e molecule(CD3E) | Hs01062241_m1 | 4331182 |
| *CD274* | CD274 molecule (CD274) | Hs00204257_m1 | 4331182 |
| *CTLA4* | cytotoxic T-lymphocyte associated protein 4(CTLA4) | Hs00175480_m1 | 4331182 |
| *HAVCR2* | hepatitis A virus cellular receptor 2(HAVCR2) | Hs00958618_m1 | 4331182 |
| *IDO1* | Indoleamine 2,3-dioxygenase 1 (IDO1) | Hs00699198_m1 | 4331182 |
| *LAG3* | lymphocyte activating 3(LAG3) | Hs00958444_g1 | 4351372 |
| *PDCD1* | programmed cell death 1(PDCD1) | Hs01550088_m1 | 4331182 |
| *PDCD1LG2* | Programmed cell death 1 ligand 2 (PDCD1LG2) | Hs00228839_m1 | 4331182 |
| *TNFRSF14* | TNF receptor superfamily member 14 (TNFRSF14) | Hs00998605_g1 | 4331182 |
| *TNFRSF9* | TNF receptor superfamily member 9 (TNFRSF9) | Hs00155512_m1 | 4331182 |
| *GAPDH* | glyceraldehyde-3-phosphate dehydrogenase(GAPDH) | Hs01548420_m1 | 4331182 |

*All probes were purchased from Applied Biosystems, Waltham, MA

| **Supplemental Table S3. Covariates of unconditional logistic regression model, multivariate Cox proportional hazard model for recurrence and survival** | | | | | | | | | | | | |  |
| --- | --- | --- | --- | --- | --- | --- | --- | --- | --- | --- | --- | --- | --- |
| Covariates | | Model for  tumor stage Coef.(95%CI)* | | *P* value | | Model for recurrence  Coef.(95%CI)** | | *P* value | | Model for  survival Coef.(95%CI)*** | | *P* value | |
| Sex | -0.04(-0.72-0.65) | | 0.92 | | 0.41 (-0.11-0.94) | | 0.12 | | -1.22 (-2.40--0.04) | | 0.04 | |  |
| Age | 0.01 (-0.02-0.05) | | 0.41 | | -0.03 (-0.05-0.002) | | 0.07 | | 0.05 (0.001-0.09) | | 0.04 | |  |
| Smoking status |  | |  | |  | |  | |  | |  | |  |
| Former | -0.12 (-0.75-0.51) | | 0.71 | | -0.06 (-0.59-0.47) | | 0.82 | | -1.05 (-1.98--0.12) | | 0.03 | |  |
| Current | -0.08 (-1.25-1.08) | | 0.89 | | 0.73 (-0.04-1.50) | | 0.06 | | 0.53 (-0.57-1.63) | | 0.35 | |  |
| BMI | -0.01 (-0.06-0.04) | | 0.62 | | -0.03 (-0.07-0.007) | | 0.11 | | -0.07 (-0.14--0.004) | | 0.04 | |  |
| Hypertension | -0.10 (-0.83-0.63) | | 0.79 | | 0.03 (-0.52-0.59) | | 0.91 | | 0.06 (-0.94-1.06） | | 0.91 | |  |
| Diabetes | 0.17 (-0.63-0.96) | | 0.68 | | -0.37 (-0.94-0.20) | | 0.2 | | -1.08 (-1.97--0.19) | | 0.02 | |  |
| Histology | NA | | NA | | 0.39 (-0.27-1.04) | | 0.25 | | 0.49 (-0.61-1.60) | | 0.38 | |  |
| Stage | NA | | NA | | 1.98 (1.42-2.54) | | 0 | | 0.92 (0.15-1.70) | | 0.02 | |  |
| Tumor grade† | NA | | NA | | 0.49 (0.30-0.67) | | 0 | | 0.27 (-0.03-0.56) | | 0.08 | |  |
| Treatment‡ | NA | | NA | | -0.04 (-0.71-0.64) | | 0.92 | | -0.14 (-1.20-0.92) | | 0.79 | |  |
| *: covariates of unconditional logistic regression model for tumor stage;  **: covariates of multivariate Cox proportional hazard model for recurrence;  ***covariates of multivariate Cox proportional hazard model for survival.  † Tumor grade based on Fuhrman criteria. ‡indicate adjuvant chemotherapy | | | | | | | | | | | | |  |

| **Supplemental Table S4. Circulating levels of soluble immune checkpoint proteins in ccRCC patients** | | | | | | | | | |
| --- | --- | --- | --- | --- | --- | --- | --- | --- | --- |
|  | **Early Stage^c^** | | |  | **Late Stage^c^** | |  |  |  |
| **Marker** | **No** |  | **Median (IQR) pg/ml** |  | **No** | **Median (IQR) pg/ml** |  | ***P* value** | **cutoff^b^** |
| sBTLA | 76 |  | 144.65 (144.65-852.02) |  | 82 | 144.65 (144.65-469.94) |  | 0.48 | 2269 |
| sCD27 | 87 |  | 350.64 (192.04-753.37) |  | 90 | 307.57 (179.93-595.62) |  | 0.47 | 1029 |
| sCD28 | 75 |  | 42.02 (42.02-149.34) |  | 76 | 42.02 (42.02-138.74) |  | 0.86 | 42.68 |
| sCD80^a^ | 83 |  | 43.58 (43.58-43.58) |  | 86 | 43.58 (43.58-43.58) |  | 0.54 | 43.58 |
| sCD137^a^ | 77 |  | 14.21 (14.21-14.21) |  | 82 | 14.21 (14.21-14.21) |  | 0.96 | 110.2 |
| sCTLA4 | 76 |  | 24.78 (9.52-61.01) |  | 80 | 33.14 (9.52-66.64) |  | 0.32 | 33.14 |
| sGITR^a^ | 79 |  | 27.20 (27.20-27.20) |  | 85 | 27.20 (27.20-27.20) |  | 0.71 | 27.2 |
| sHVEM^a^ | 85 |  | 18.55 (18.55-18.56) |  | 85 | 18.55 (18.55-18.56) |  | 0.45 | 18.55 |
| sIDO | 76 |  | 4.37 (4.37-18.33) |  | 75 | 4.37 (4.37-12.96) |  | 0.73 | 4.37 |
| sLAG3 | 86 |  | 139.98 (81.58-216.34) |  | 90 | 160.55 (103.70-206.76) |  | 0.18 | 258 |
| sPD1^a^ | 88 |  | 7.13 (7.13- 7.13) |  | 91 | 7.13 (7.13- 7.13) |  | - | - |
| sPDL1 | 73 |  | 7.26 (3.64-21.67) |  | 75 | 7.85 (3.64-19.72) |  | 0.94 | 13.37 |
| sPDL2 | 85 |  | 1381.38 (48.71-5328.93) |  | 87 | 1348.71(48.71-6132.34) |  | 0.92 | 3032 |
| sTIM3 | 87 |  | 3628.83 (2713.02-5176.08) |  | 90 | 4226.43 (2962.89-5696.84) |  | 0.16 | 5908 |
| Abbreviations: ccRCC, clear cell renal cell carcinoma; IQR, interquartile range.  ^a^ CD137, HVEM, GITR, CD80 and PD1 level demonstrated few variations, thus these markers were not included in the subsequent analysis.  ^b^ Levels of all soluble biomarkers and immune checkpoint genes were dichotomized into low- and high-level groups using a logistic regression spline model *(12)*.  ^c^ Early stage indicates stage I&II disease, Late stage indicate stage III disease, the staging criteria according to NCCN guideline 2019 v.2.0. | | | | | | | | |  |

| **Supplemental Table S5. Association with sTIM3 with death risk of ccRCC patients stratified by clinical stage** | | | | | |
| --- | --- | --- | --- | --- | --- |
| **Protein name** | **Early stage (I & II)** | |  | **Late stage (III)** | |
| **High *vs* low**^*^ | **Adjusted HR (95%CI)**^†^ | ***P* value** |  | **Adjusted HR (95%CI)**^†^ | ***P* value** |
| sTIM3 | 1 (reference) |  |  | 1 (reference) |  |
|  | **36.14 (3.733-350)** | **1.95E-03^#^** |  | 1.62 (0.60-4.41) | 0.34 |
| Abbreviations: ccRCC, clear cell renal cell carcinoma; HR, hazard ratio; CI confidence interval.  Significant values in bold font.  * High- and low-level groups dichotomized by the logistic regression spline model (12).  † Adjusted by age, gender, smoking, BMI, diabetes, hypertension, histology, grade, and treatment.  # Significant after Bonferroni adjustment for multiple testing. | | | | | |

| **Supplemental Table S6. Risk scores of soluble immune checkpoint biomarkers for ccRCC survival** | | | | | | |
| --- | --- | --- | --- | --- | --- | --- |
| **Risk score *** | **Dead** | **Alive** | **HR (95% CI)**† | ***P* value** | **MST (month)** | ***P* log-rank** |
| Low | 11 (9.02) | 111 (90.98) | 1 (reference) |  | - |  |
| Medium | 8 (30.77) | 18 (69.23) | 3.29 (1.14-9.52) | 0.028 | - |  |
| High | 14 (41.18) | 20 (61.11) | 12.88 (3.62-45.78) | 7.88E-05 | 22.4 | 5.14E-11 |
| Abbreviations: ccRCC, clear cell renal cell carcinoma; HR, hazard ratio; CI, confidence interval; MST, median survival time.  * Risk groups were divided into tertiles based on levels of sBTLA and sTIM3.  † Adjusted by age, gender, smoking, BMI, diabetes, hypertension, histology, grade, stage and treatment.  (-) MST greater than follow-up time. | | | | | | |

| **Supplemental Table S7: The correlation between circulating checkpoint level and T cell functions** | | | | | | | | | | | | |
| --- | --- | --- | --- | --- | --- | --- | --- | --- | --- | --- | --- | --- |
|  |  | ***CD8A*** | |  | **CYT score 1†** | |  | **CYT score 2†** | |  | ***IFNG*** | |
| **Markers** | ***N*** | **rho** | ***P*** |  | **rho** | ***P*** |  | **rho** | ***P*** |  | **rho** | ***P*** |
| sTIM3 | 46 | -0.17 | 0.26 |  | -0.14 | 0.35 |  | 0.00 | 0.99 |  | -0.10 | 0.50 |
| **sCD28** | **38** | -0.25 | 0.14 |  | **-0.33** | **0.05*** |  | **-0.33** | **0.05*** |  | -0.07 | 0.67 |
| sCD137 | 40 | 0.16 | 0.33 |  | 0.04 | 0.80 |  | 0.04 | 0.80 |  | -0.03 | 0.83 |
| sCD27 | 45 | -0.06 | 0.72 |  | 0.01 | 0.96 |  | 0.17 | 0.27 |  | 0.15 | 0.32 |
| sCTLA4 | 39 | -0.05 | 0.75 |  | -0.08 | 0.65 |  | -0.02 | 0.89 |  | 0.28 | 0.08 |
| sHVEM | 43 | -0.06 | 0.71 |  | -0.10 | 0.52 |  | -0.10 | 0.51 |  | -0.12 | 0.44 |
| sIDO | 35 | -0.06 | 0.73 |  | -0.05 | 0.79 |  | -0.11 | 0.52 |  | 0.13 | 0.46 |
| **sLAG3** | **46** | **-0.35** | **0.02** |  | **-0.31** | **0.04** |  | -0.25 | 0.09 |  | -0.21 | 0.17 |
| sBTLA | 39 | -0.05 | 0.76 |  | -0.09 | 0.59 |  | 0.07 | 0.68 |  | -0.05 | 0.75 |
| sPD1 | 46 | 0.08 | 0.61 |  | 0.00 | 0.97 |  | -0.08 | 0.59 |  | -0.15 | 0.33 |
| sPDL1 | 36 | 0.25 | 0.15 |  | 0.29 | 0.09 |  | 0.45 | 0.01 |  | **0.51** | **1.31E-03** |
| sPDL2 | 43 | 0.05 | 0.76 |  | 0.11 | 0.49 |  | 0.18 | 0.24 |  | 0.19 | 0.22 |

* Before rounding, *P*=0.0464 and *P*=0.0459, respectively.

^1^ Cytolytic score based on *GZMA* and *PRF1* tissue expression.

^2^ Cytolytic score based on *GZMB* and *PRF1* tissue expression.

| **Supplemental Table S8. Tumor expression of immune checkpoint genes and association with ccRCC overall survival in the MDACC and TCGA cohorts** | | | | | | | | | | | | |
| --- | --- | --- | --- | --- | --- | --- | --- | --- | --- | --- | --- | --- |
|  |  | **MDACC cohort (*n*=47)** | | | | |  | **TCGA cohort (*n*=382)^#^** | | | | |
| **Gene** | **Expression** | **Dead** | **Alive** | **HR (95%CI)*** | ***P* value** | ***P* log-rank** |  | **Dead** | **Alive** | **HR (95%CI) ^†^** | ***P* value** | ***P* log-rank** |
| *BTLA* | low | 7 (20.59) | 27 (79.41) | 1 (reference) |  |  |  | 49(25.65) | 142(74.35) | 1(reference) |  |  |
|  | high | 3 (23.08) | 10 (76.92) | 1.41 (0.27-7.46) | 0.69 | 0.25 |  | 43(22.51) | 148(77.49) | 0.73 (0.47-1.14) | 0.17 | 0.35 |
| *CD27* | low | 6(25.00) | 18(75.00) | 1 (reference) |  |  |  | 39(20.42) | 152(79.58) | 1(reference) |  |  |
|  | high | 4(17.39) | 19(82.61) | 0.27 (0.03-2.76) | 0.27 | 0.93 |  | 53(27.75) | 138(72.25) | 1.00(0.65-1.56) | 0.97 | 0.21 |
| *CD28* | low | 6(20.69) | 23(79.31) | 1 (reference) |  |  |  | 41(21.47) | 150(78.53) | 1(reference) |  |  |
|  | high | 4(22.22) | 14(77.78) | 1.08 (0.22-5.16) | 0.93 | 0.55 |  | 51(26.70) | 140(73.30) | 1.11 (0.72-1.71) | 0.63 | 0.47 |
| *CD274* | low | 7 (24.14) | 22 (75.86) | 1 (reference) |  |  |  | 59(30.89) | 132(69.11) | 1(reference) |  |  |
|  | high | 3 (16.67) | 15 (83.33) | 0.77 (0.13-4.59) | 0.78 | 0.48 |  | 33(17.28) | 158(82.72) | 0.51(0.33-0.80) | **3.23E-03** | **6.70E-04** |
| *CTLA4* | low | 7(21.88) | 25(78.13) | 1 (reference) |  |  |  | 39(20.42) | 152(79.58) | 1(reference) |  |  |
|  | high | 3(20.00) | 12(80.00) | 0.80(0.14-4.58) | 0.80 | 0.66 |  | 53(27.75) | 138(72.25) | 1.22 (0.78-1.89) | 0.38 | **0.05** |
| *HAVCR2* | low | 4(15.38) | 22(84.62) | 1 (reference) |  |  |  | 52(27.23) | 139(72.77) | 1(reference) |  |  |
|  | high | 6(28.57) | 15(71.43) | 5.79 (1.00-33.7) | **0.05** | 0.32 |  | 40(20.94) | 151(79.06) | 0.59(0.39-0.91) | **0.02** | 0.08 |
| *IDO1* | low | 5 (20.00) | 20 (80.00) | 1 (reference) |  |  |  | 41(21.47) | 150(78.53) | 1(reference) |  |  |
|  | high | 5 (22.73) | 17 (77.27) | 1.10 (0.20-6.14) | 0.91 | 0.54 |  | 51(26.70) | 140(73.30) | 0.94 (0.62-1.44) | 0.79 | 0.45 |
| *LAG3* | low | 6 (22.22) | 21 (77.78) | 1 (reference) |  |  |  | 35(18.32) | 156(81.68) | 1(reference) |  |  |
|  | high | 4 (20.00) | 16 (80.00) | 1.40 (0.20-10.0) | 0.74 | 0.70 |  | 57(29.84) | 134(70.16) | 1.14 (0.73-1.78) | 0.56 | 0.03 |
| *PDCD1* | low | 7 (21.21) | 26 (78.79) | 1 (reference) |  |  |  | 41(21.47) | 150(78.53) | 1(reference) |  |  |
|  | high | 3 (21.43) | 11 (78.57) | 0.98 (0.18-5.17) | 0.98 | 0.68 |  | 51(26.70) | 140(73.30) | 0.97 (0.62-1.51) | 0.89 | 0.28 |
| *PDCD1LG2* | low | 3 (10.34) | 26 (89.66) | 1 (reference) |  |  |  | 51(26.70) | 140(73.30) | 1(reference) |  |  |
|  | high | 7 (38.89) | 11 (61.11) | 8.62 (1.47-50.7) | **0.02** | **0.01** |  | 41(21.47) | 150(78.53) | 0.69 (0.44-1.06) | 0.09 | 0.17 |
| *TNFRSF14* | low | 6(20.69) | 23(79.31) | 1(reference) |  |  |  | 47(24.61) | 144(75.39) | 1(reference) |  |  |
|  | high | 4(22.22) | 14(77.78) | 3.85 (0.37-40.1) | 0.26 | 0.65 |  | 45(23.56) | 146(76.44) | 0.92 (0.60-1.42) | 0.72 | 0.79 |
| *TNFRSF9* | low | 5(21.74) | 18(78.26) | 1(reference) |  |  |  | 46(24.08) | 145(75.92) | 1(reference) |  |  |
|  | high | 5(20.83) | 19(79.17) | 0.39 (0.05-2.85) | 0.35 | 0.53 |  | 46(24.08) | 145(75.92) | 0.73 (0.47-1.16) | 0.14 | 0.95 |
| * adjusted by age, gender, smoking, BMI, diabetes, hypertension, histology, grade and stage.  † adjusted by age, sex, grade, stage.  ^#^ Number of patients analyzed is less than total due to missing clinical outcome information. | | | | | | | | | | | | |

**Supplemental Figures**


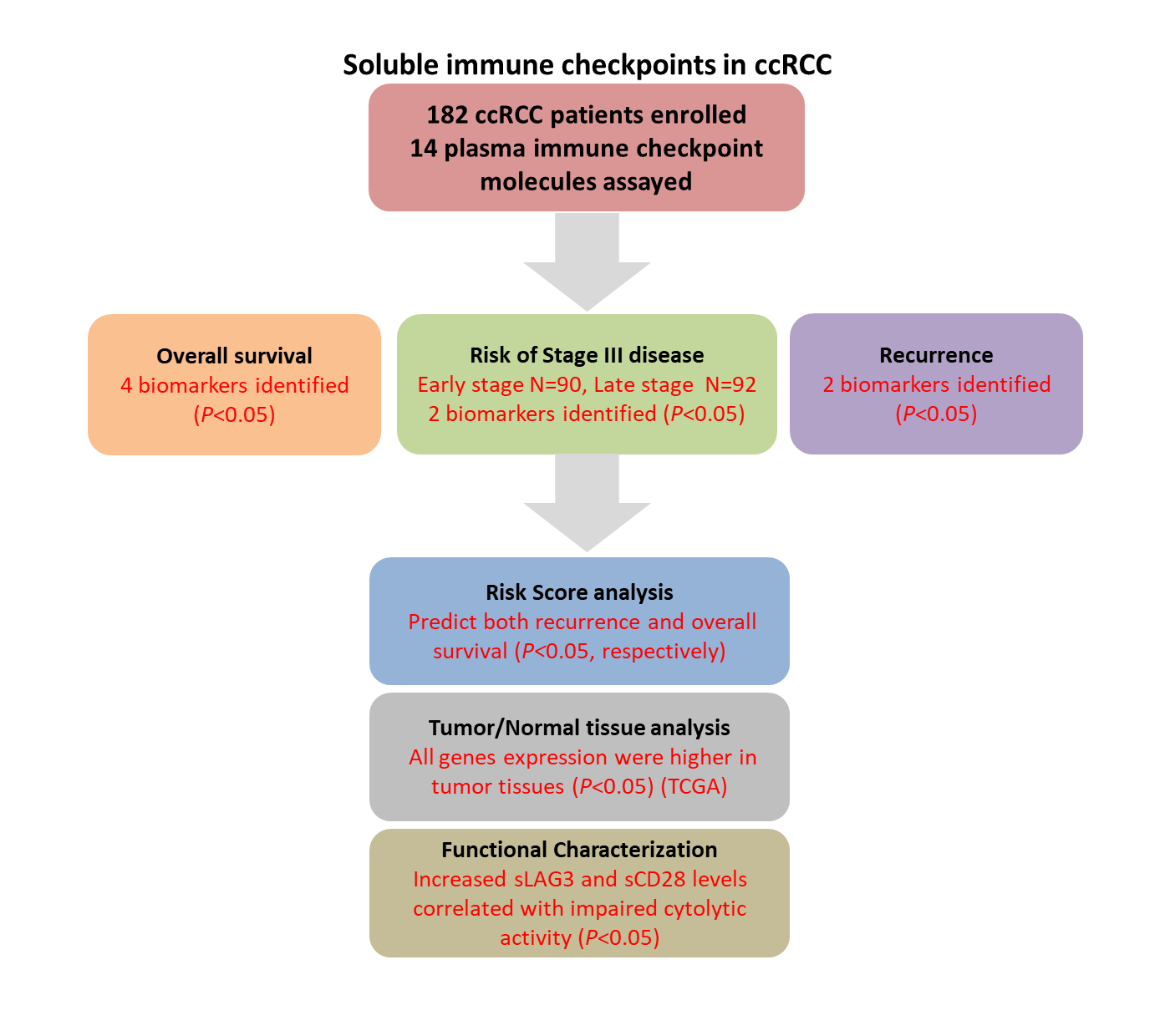


**Supplemental Figure S1.** Schematic design of the study


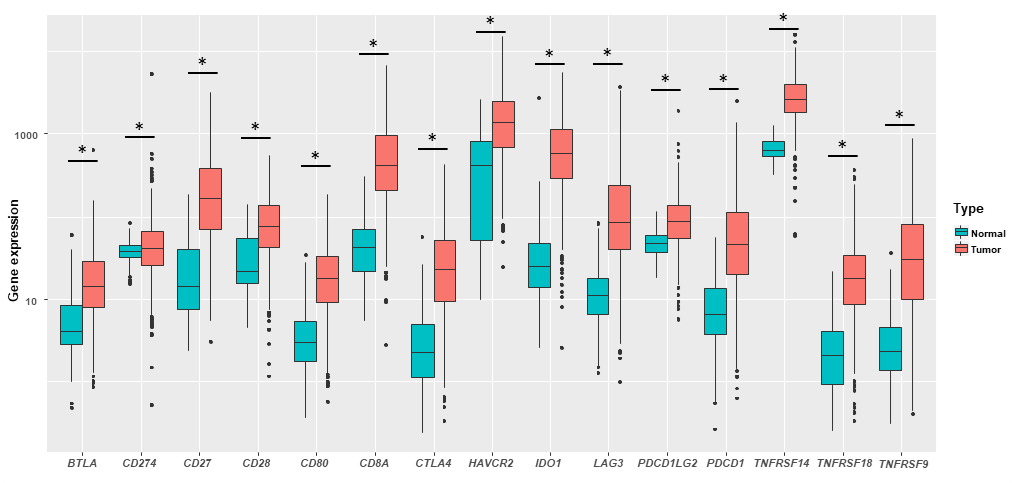


**Supplemental Figure S2**. Tumor and normal tissue expression of immune checkpoint genes in ccRCC patients derived from TCGA database. Maroon boxplot indicates tumor tissues (*N*=533), and green boxplot indicates normal tissues (*N*=72). Wilcoxon rank sum test was used in the comparison analysis. *indicates *P*<0.05.
